# Synergistic Anti-quorum Sensing and Anti-biofilm Activities of Crude Terminalia catappa Leaf Extract Combined With Ciprofloxacin Against Pseudomonas aeruginosa

**DOI:** 10.7759/cureus.103643

**Published:** 2026-02-15

**Authors:** Renz Marion L Ricafrente, Analiza P Malalay

**Affiliations:** 1 College of Pharmacy, Our Lady of Fatima University, Quezon City, PHL; 2 College of Pharmacy, University of Perpetual Help System Laguna, Biñan, PHL

**Keywords:** antibiotic adjuvant, biofilm formation, checkerboard assay, ciprofloxacin, pseudomonas aeruginosa, quorum sensing inhibition, terminalia catappa

## Abstract

The increasing prevalence of biofilm-associated infections and antimicrobial resistance highlights the need for alternative and complementary therapeutic strategies. This study investigated the anti-quorum-sensing, anti-biofilm, and antibacterial activities of *Terminalia catappa* ethanolic crude leaf extract combined with ciprofloxacin against *Pseudomonas aeruginosa* BIOTECH 1335 using an in vitro quantitative experimental design. The crude extract was obtained from fresh leaves, yielding 2.46% (2.46 g/100 g), and phytochemical screening revealed the presence of flavonoids, tannins, cardiac glycosides, reducing sugars, and fixed oils, while alkaloids and anthraquinone glycosides were absent. Fourier transform infrared spectroscopy with attenuated total reflectance (FTIR-ATR) analysis was performed using an IRPrestige-21 FTIR spectrophotometer (Shimadzu Corporation, Kyoto, Japan) equipped with a MIRacle ATR accessory (PIKE Technologies, Madison, WI, USA), confirming functional groups associated with phenolic and flavonoid compounds. Biofilm inhibition assays showed that the extract alone achieved up to 67.23% inhibition at 64 µg/mL. Ciprofloxacin demonstrated an inverse dose-response pattern in biofilm inhibition, wherein higher concentrations exhibited lower inhibitory effects, an atypical finding of potential mechanistic relevance. The combined treatment produced enhanced inhibition at defined concentration ranges, with the highest biofilm inhibition (72.94%) observed at 64 µg/mL extract and 0.0625 µg/mL ciprofloxacin. Quorum-sensing assays showed dose-dependent reductions in swarming and swimming motility across treatments. Checkerboard analysis revealed concentration-dependent synergistic interactions (∑FIC ≤ 0.5) specifically at low-to-moderate extract concentrations (16-32 µg/mL) combined with sub-inhibitory ciprofloxacin doses (0.015625-0.03125 µg/mL), while higher-dose combinations demonstrated predominantly additive effects. Two-way ANOVA confirmed significant main effects of extracts and ciprofloxacin concentrations. Overall, the findings demonstrate that *Terminalia catappa *crude extract can potentiate the antibacterial and anti-biofilm activity of ciprofloxacin at specific concentration combinations, supporting the potential of plant-antibiotic combination strategies as a multi-targeted approach against biofilm-related infections and antimicrobial resistance.

## Introduction

In recent years, there has been increasing scientific interest in natural plant extracts as alternative or complementary antimicrobial agents due to the rapid rise of antimicrobial resistance. Medicinal plants are recognized as rich sources of bioactive compounds such as flavonoids, tannins, phenolics, and alkaloids, which exhibit antibacterial, antioxidant, and anti-inflammatory properties. *Terminalia catappa*, commonly known as tropical almond, has gained considerable attention because of its wide traditional use and documented pharmacological activities, including antibacterial effects against a range of pathogenic microorganisms [1,2]. Its incorporation into commercial formulations such as antibacterial soaps further supports its functional antimicrobial potential [3].

However, the effective management of bacterial infections remains challenged by biofilm formation, a major contributor to treatment failure and persistent infections. *Pseudomonas aeruginosa* is a clinically important opportunistic pathogen known for its intrinsic antibiotic resistance, strong biofilm-forming capacity, and ability to cause chronic infections in immunocompromised individuals. Biofilms are structured microbial communities embedded in an extracellular polymeric matrix that significantly reduces antibiotic penetration and enhances bacterial survival [4]. This characteristic makes *P. aeruginosa* infections particularly difficult to eradicate using conventional antibiotic monotherapy.

In addition, *P. aeruginosa* regulates biofilm development and virulence through quorum sensing, a bacterial communication system that coordinates gene expression in response to population density. Quorum sensing controls critical behaviors such as motility, toxin production, and biofilm maturation, thereby contributing to increased pathogenicity and antimicrobial resistance [5]. Targeting quorum sensing has emerged as a promising anti-virulence strategy, as it interferes with bacterial coordination without exerting strong selective pressure for resistance.

Plant-derived polyphenols such as flavonoids and tannins have been reported to interfere with quorum-sensing pathways through multiple mechanisms. In Gram-negative bacteria such as *P. aeruginosa, *quorum sensing is primarily mediated by acyl-homoserine lactone (AHL) signaling molecules regulated by LuxI/LuxR homologs, including the Las and Rhl systems [5]. Certain flavonoids have been shown to competitively or non-competitively bind to quorum-sensing receptors such as LasR, disrupt AHL-mediated gene activation, and suppress virulence factor production and biofilm formation [5]. These findings suggest that plant-derived polyphenolic compounds may attenuate pathogenic behaviors by targeting quorum-sensing-regulated pathways rather than directly exerting bactericidal effects.

Although *T. catappa* has been shown to possess antibacterial and antioxidant activities, most investigations have primarily focused on its direct antimicrobial effects. Limited research has specifically evaluated its anti-quorum-sensing activity in *P. aeruginosa*, particularly with respect to quorum-sensing-associated phenotypes such as motility and biofilm formation. Recent research suggests that plant-derived compounds can act as antibiotic adjuvants by enhancing drug efficacy, lowering required antibiotic doses, and improving activity against biofilm-associated bacteria [6]. However, evidence supporting the synergistic interaction between *T. catappa *crude extract and ciprofloxacin using systematic checkerboard analysis against *P. aeruginosa *remains insufficient. Therefore, a clear gap remains in evaluating its potential role as an antibiotic adjuvant targeting both bacterial growth and virulence-associated behaviors. 

Therefore, this study aims to investigate the anti-quorum-sensing, anti-biofilm, and antibacterial effects of *T. catappa* crude leaf extract in combination with ciprofloxacin against *P. aeruginosa*. Specifically, it evaluates microbial growth inhibition, biofilm suppression, and quorum-sensing disruption of the combined treatment compared to each agent alone. The findings of this research are expected to contribute to the development of plant-antibiotic synergistic strategies and provide scientific support for alternative approaches in addressing biofilm-associated infections and antimicrobial resistance.

## Materials and methods

To obtain the necessary data for this study, a quantitative experimental research design was employed. Quantitative research is a method that relies on measuring variables using numerical systems, analyzing these measurements with statistical models, and reporting the relationships and associations among the studied variables [7]. Specifically, this study utilized an in vitro experimental design to investigate the antibacterial, anti-biofilm, and quorum-sensing-associated phenotypic effects of *T. catappa* crude ethanolic leaf extract, ciprofloxacin, and their combination against *P. aeruginosa* BIOTECH 1335. Experimental research involves a structured and controlled approach that allows precise manipulation and observation of variables, making it suitable for evaluating the interactions between the plant extract and the antibiotic [8].

The test organism used in this study was *P. aeruginosa* BIOTECH 1335, obtained from the Philippine National Collection of Microorganisms (PNCM) at the University of the Philippines Los Baños (UPLB). Bacterial cultures were prepared on nutrient agar under standard laboratory conditions and standardized for use in subsequent assays.

The plant material, *T. catappa* leaves, was collected from Brgy. Poblacion, Guinayangan, Quezon, identified and authenticated at the University of the Philippines, washed, oven-dried at 50 °C for two days using a drying oven (BIOBASE BOV-V70F, BIOBASE Group, Jinan, Shandong, China), and ground into a fine powder. A total of 100 g of oven-dried leaf powder was subjected to extraction via maceration with 95% ethanol at a ratio of 1:4 for one week, followed by filtration and concentration using a rotary evaporator (EYELA N-1210 Series, Tokyo Rikakikai Co., Ltd., Tokyo, Japan) at 50 °C [9]. The extraction yielded 2.46 g of crude extract, corresponding to a percentage yield of 2.46% (w/w). All experiments were conducted using an extract derived from a single extraction batch. The crude extract was stored at 4 °C for further use.

Phytochemical screening of the crude extract was conducted using standard qualitative methods to detect secondary metabolites such as alkaloids, flavonoids, tannins, cardiac glycosides, anthraquinone glycosides, reducing sugars, and fixed oils and fats [10]. Fourier transform infrared spectroscopy with attenuated total reflectance (FTIR-ATR) was performed to identify functional groups present in the extract [11].

The minimum inhibitory concentration (MIC) of both the crude extract and ciprofloxacin was determined using the broth microdilution method in 96-well plates, with optical density readings at 630 nm obtained using a microplate reader (ALLSHENG AMR-100, Hangzhou, China). The MIC was defined as the lowest concentration showing no visible bacterial growth and an optical density comparable to the negative control after 24 hours of incubation at 37 °C. Sub-MIC concentrations were then prepared for subsequent biofilm inhibition, quorum sensing motility, and checkerboard synergy assays to ensure that observed effects were due to anti-virulence activity rather than bactericidal action [12,13].

Biofilm inhibition was assessed using the crystal violet assay. Quorum-sensing-associated motility was evaluated using swimming and swarming assays. Swimming motility was performed on semi-solid nutrient agar containing 0.3% agar, while swarming motility was assessed on nutrient agar containing 0.5% agar. Sub-MIC concentrations of the extract and/or ciprofloxacin were incorporated into the medium prior to solidification. Plates (90 mm in diameter) were inoculated with 2 µL of standardized *P. aeruginosa* suspension and incubated at 37 °C for 24 hours. Motility zones were measured in millimeters and compared with untreated controls to determine percentage reduction [14].

Synergistic interactions between *T. catappa* crude extract and ciprofloxacin were evaluated using the checkerboard microdilution method. Interactions were interpreted based on the Fractional Inhibitory Concentration Index (∑FIC) as synergistic (≤0.5), additive (>0.5-1.0), indifferent (>1.0-4.0), or antagonistic (>4.0) [15].

All experiments were conducted in triplicate, with at least three independent biological replicates. Appropriate controls, including sterility, solvent, growth, and positive controls, were included in each assay. Data were analyzed using analysis of variance (ANOVA) with Tukey’s post hoc test for biofilm and motility assays and two-way ANOVA for checkerboard synergy analysis, with statistical significance set at p < 0.05. Percentage biofilm inhibition, motility reduction, and FIC values were calculated using standard formulas. This methodological framework enabled a comprehensive evaluation of the antibacterial, anti-biofilm, and quorum-sensing-associated phenotypic effects of *T. catappa *crude extract alone and in combination with ciprofloxacin against *P. aeruginosa *BIOTECH 1335.

## Results

The yield of the ethanolic crude extract obtained from *T. catappa *leaves is summarized in Table 1. From 100 g fresh *T. catappa* leaves, a total of 2.46 g of ethanolic crude extract was obtained after maceration with 95% ethanol and evaporation to dryness. This corresponds to a 2.46% yield based on the initial fresh weight.

**Table 1:** Yield of ethanolic crude extract obtained from Terminalia catappa leaves

Parameter	Fresh weight (g)	Ethanolic crude extract after evaporation (g)	% yield
*Terminalia catappa* leaves	100 g	2.46 g	2.46 %

Qualitative phytochemical screening

The qualitative phytochemical screening results of the ethanolic leaf extract of *T. catappa* are presented in Table 2. Both Mayer’s and Dragendorff’s tests yielded negative results, indicating the absence of alkaloids. The Bate-Smith and Metcalf test produced a red coloration, while the Wilstatter cyanidin test showed an orange-red color, confirming the presence of flavonoids. The ferric chloride test indicated a dark-colored precipitate, signifying the presence of tannins. 

**Table 2:** Qualitative phytochemical screening results of the ethanolic leaf extract of Terminalia catappa

Phytochemical test	Observation	Result
Test for alkaloids		
Dragendorff’s test	No precipitate formed	Negative
Mayer’s test	No precipitate formed	Negative
Test for flavonoids		
Bate-Smith and Metcalf test	Formation of red color	Positive
Wilstatter cyanidin test	Formation of orange-red color	Positive
Test for tannins		
Ferric chloride test	Formation of dark-colored precipitate	Positive
Test for cardiac glycosides		
Keller-Kiliani test	Brownish color at the interface	Positive
Test for anthraquinone glycosides		
Borntrager’s test	No color formation	Negative
Test for reducing sugars		
Fehling’s test	Brick-red precipitate formed	Positive
Test for fixed oils and fats		
Spot test	Greasy appearance after drying	Positive

A brownish color was observed at the interface in the Keller-Kiliani test, indicating the presence of cardiac glycosides, while the Borntrager’s test showed no color formation, suggesting the absence of anthraquinone glycosides. The Fehling’s test resulted in a brick-red precipitate, confirming the presence of reducing sugars. Finally, the filter paper test exhibited a greasy appearance after drying, which confirmed the presence of fixed oils and fats in the ethanolic extract. 

FTIR analysis and interpretation

**Figure 1:**
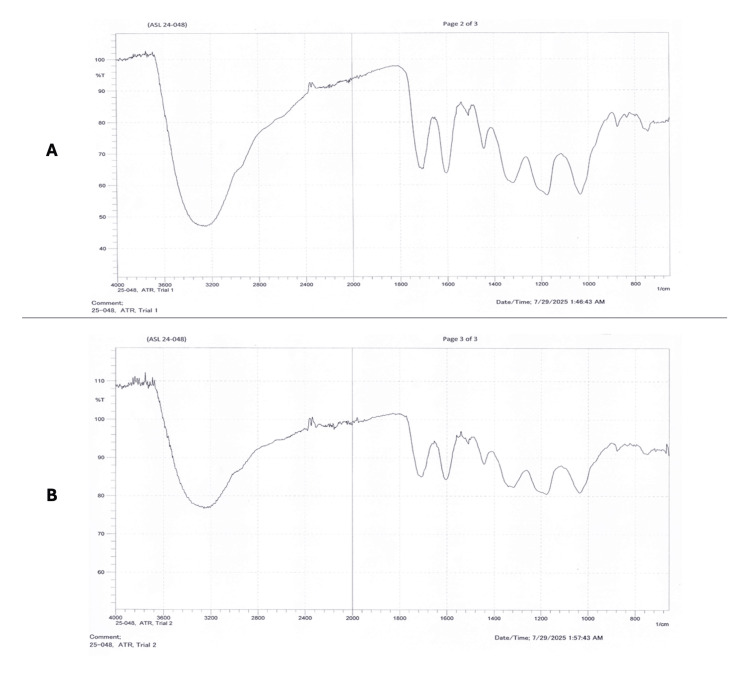
FTIR-ATR spectra of the ethanolic leaf extract of Terminalia catappa (A) Fourier transform infrared spectroscopy with attenuated total reflectance (FTIR-ATR) spectrum obtained from Trial 1. (B) FTIR-ATR spectrum obtained from Trial 2. Image obtained directly from the FTIR-ATR instrument software following analysis of the ethanolic leaf extract samples.

The FTIR-ATR spectra obtained from two independent trials of the ethanolic leaf extract of* T. catappa *are shown in Figure 1. The sample was analyzed using Fourier transform infrared spectroscopy (FTIR) in the attenuated total reflectance (ATR) mode to identify the major functional groups present. Two independent trials were conducted to assess reproducibility, and the resulting spectra were compared for consistency. 

The ATR-FTIR spectra obtained from Trial 1 and Trial 2 showed highly consistent absorption patterns, confirming the reproducibility of the measurement. Both spectra displayed a broad and intense band around 3300-3200 cm^-1^, characteristic of hydrogen-bonded O-H stretching. This broad absorption extends into the 2900 cm^-1^ region, suggesting overlapping contributions from aliphatic C-H stretching. A moderately strong band at approximately 1720 cm^-1^ indicates the presence of carbonyl (C=O) functionalities. Several well-defined peaks between 1650-1500 cm^-1^ correspond to aromatic C=C stretching and/or amide-related vibrations. The strong cluster of absorptions between 1300-1000 cm^-1^ is attributed to C-O and C-O-C stretching modes typical of alcohols, esters, ethers, or polysaccharide-derived structures. Additional peaks below 1000 cm^-1^ fall within the fingerprint region and likely represent aromatic C-H bending or other complex vibrational modes. Overall, the spectral features confirm that the sample contains hydroxyl, carbonyl, aliphatic, aromatic/amide, and C-O/C-O-C functional groups. 

These findings indicate that *T. catappa* leaves contain multiple phytochemical groups such as flavonoids, tannins, glycosides, reducing sugars, and fixed oils, which are known to contribute to its pharmacologic properties. 

Anti-biofilm activity of *T. catappa* leaf extract

The OD_630 _values and corresponding percentage biofilm inhibition for each treatment condition are summarized in Table 3. The anti-biofilm activity of the crude extract of *T. catappa*, both as a single agent and in combination with ciprofloxacin, was assessed using a microliter plate biofilm assay. The experiment was done in triplicates and optical density (OD 630) was measured to quantify biofilm formation. Percentage inhibition was computed relative to the untreated negative control. Background correction was already accounted for in all the wells in the experiment setup based on the protocol. 

**Table 3:** Summary of OD630 and percent biofilm inhibition (mean ± SD)

Condition	OD630 (mean ± SD)	% biofilm inhibition (mean ± SD)
Negative control (bacteria only)	0.992 ± 0.023	-0.00 ± 2.34
Blank/ Media only	0.033 ± 0.002	96.64 ± 0.15
Solvent control (1% DMSO + bacteria)	0.990 ± 0.005	0.17 ± 0.50
Positive control (Ciprofloxacin 0.125 µg/mL + bacteria)	0.609 ± 0.034	38.55 ± 3.44
Cip 0.015625 µg/mL (1/8 MIC)	0.405 ± 0.041	59.19 ± 4.14
Cip 0.03125 µg/mL (1/4 MIC)	0.609 ± 0.034	38.55 ± 3.44
Cip 0.0625 µg/mL (1/2 MIC)	0.767 ± 0.027	22.66 ± 2.71
Extract 16 µg/mL (1/2 MIC)	0.688 ± 0.014	30.59 ± 1.36
Extract 32 µg/mL (1/4 MIC)	0.481 ± 0.014	51.50 ± 1.43
Extract 64 µg/mL (1/2 MIC)	0.325 ± 0.013	67.23 ± 1.31
(Comb) Extract 16 + Cip 0.015631	0.594 ± 0.007	40.07 ± 0.72
(Comb) Extract 16 + Cip 0.03125	0.535 ± 0.009	46.05 ± 0.90
(Comb) Extract 16 + Cip 0.0625	0.498 ± 0.012	49.75 ± 1.24
(Comb) Extract 32 + Cip 0.015628	0.455 ± 0.017	54.12 ± 1.71
(Comb) Extract 32 + Cip 0.03125	0.404 ± 0.016	59.26 ± 1.57
(Comb) Extract 32 + Cip 0.0625	0.376 ± 0.019	62.12 ± 1.90
(Comb) Extract 64 + Cip 0.015625	0.350 ± 0.009	64.67 ± 0.92
(Comb) Extract 64 + Cip 0.03125	0.307 ± 0.009	69.08 ± 0.86
(Comb) Extract 64 + Cip 0.0625	0.268 ± 0.014	72.94 ± 1.41

**Figure 2:**
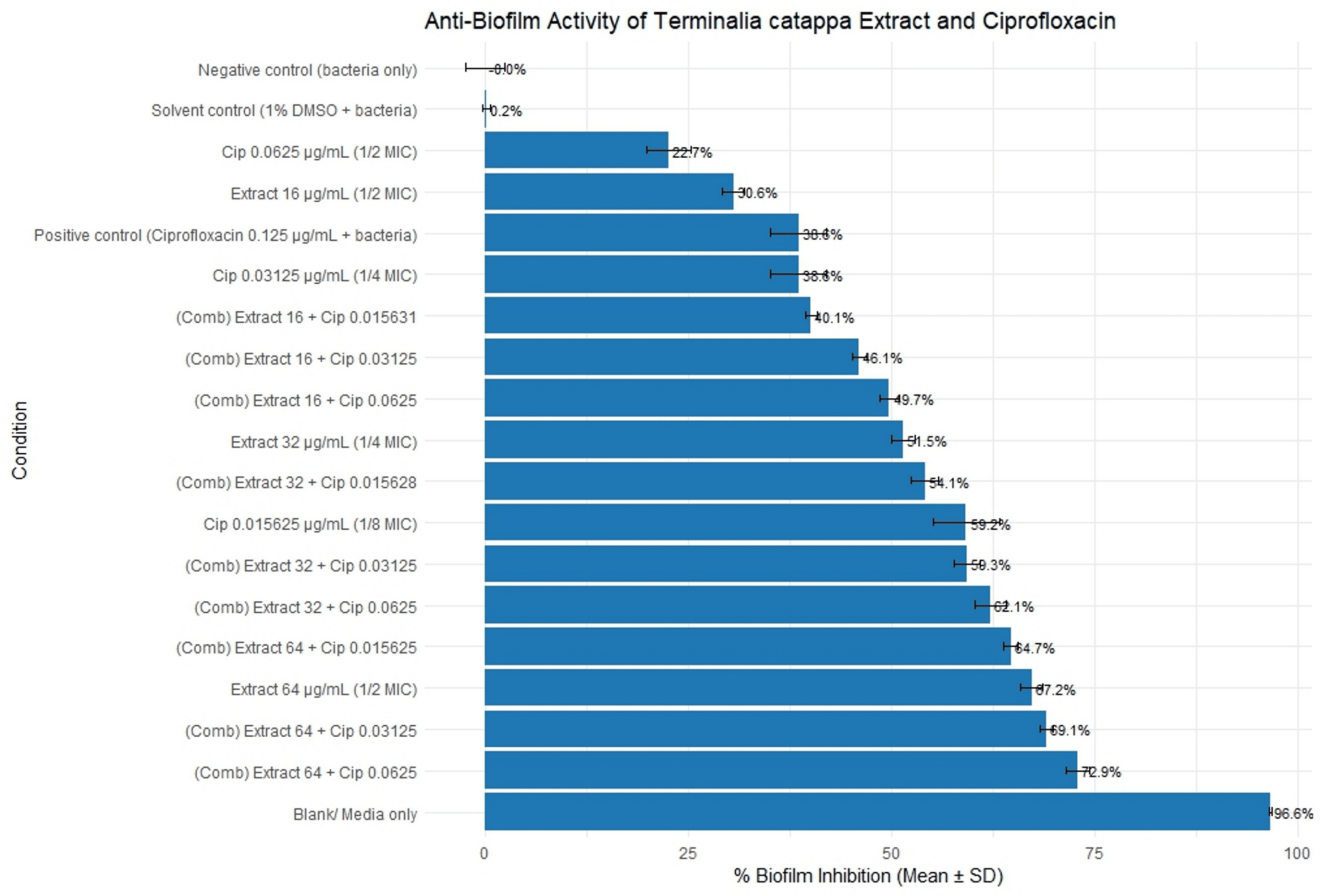
Effect of sub-MIC concentrations of Terminalia catappa crude leaf extract, ciprofloxacin, and their combinations on the biofilm formation of Pseudomonas aeruginosa BIOTECH 1335 Percentage biofilm inhibition of *Pseudomonas aeruginosa* following treatment with sub-MIC concentrations of *Terminalia catappa* crude leaf extract, ciprofloxacin, and their combinations. Bars represent mean percentage inhibition, and error bars indicate standard deviation (SD). Negative control (bacteria only), solvent control (1% DMSO + bacteria), and blank (media only) are included for comparison. Image created by the authors with Microsoft Excel (Microsoft Corp., USA)

The effects of sub-MIC concentrations of *T. catappa *extract, ciprofloxacin, and their combinations on biofilm formation are illustrated in Figure 2. In the biofilm assay, assessing the reliability of the experiment setup, the blank wells (media only) measure a mean OD of 0.03, which corresponds to 96.64% inhibition, which is expected considering that the well does not contain bacteria; the optical density observed was negligible and a reliable baseline for the experiment. Moreover, the negative control showed maximum biofilm formation, which is expected since this is the untreated control. The positive control (ciprofloxacin 0.125 µg/mL + bacteria) served as the standard and had a 38.55% biofilm inhibition. 

Biofilm inhibition by ciprofloxacin, *T. catappa *crude extract, and their combinations was evaluated. The extract demonstrated dose-dependent inhibition, with the highest tested concentration (64 µg/mL) reducing biofilm formation by 67.23 ± 1.31%. By contrast, ciprofloxacin exhibited an inverse dose-response pattern, wherein the lowest sub-MIC concentration (0.015625 µg/mL, ⅛ MIC) achieved the highest inhibition at 59.19 ± 4.14%, while higher concentrations produced lower inhibition. The highest inhibition among all treatments was observed in the combination of *T. catappa extract *(64 µg/mL) and ciprofloxacin (0.0625 µg/mL), which achieved 72.94 ± 1.41% biofilm inhibition.

The bar plot illustrates the percentage inhibition exhibited by each treatment condition, arranged from least to highest inhibition, and how varied these values were across the replicates of the experiment. 

From this plot, the lower end of the inhibition spectrum was the untreated or negative control and the solvent control (1% DMSO + bacteria), which demonstrated negligible inhibition establishing that untreated cells were able to form robust biofilms. Following this, the lowest inhibition values were observed from sub-MIC concentrations of both ciprofloxacin and *T. catappa*. The lowest inhibition was at 22.7% from ciprofloxacin at 0.0625 µg/mL and followed by extract 16 µg/mL at 30.62%, which represents the mildest anti-biofilm activities among the tested agents.

The highest among the single agents is from the highest sub-MIC of *T. catappa*, which is at 64 µg/mL and achieved 67.23% inhibition, surpassing all single agent extracts. The most effective conditions were consistently the extract-ciprofloxacin combinations, particularly those involving the highest extract dose. The strongest inhibition was observed when extracting 64 µg/mL + ciprofloxacin 0.0625 µg/mL, which produced 72.94% inhibition, representing the maximum anti-biofilm performance among all treatments. 

The results of the one-way ANOVA assessing differences in OD_630 _values among treatment groups are presented in Table 4. At the 0.05 level of significance, the analysis revealed a statistically significant difference among the treatment groups, indicating that at least one treatment exhibited a significantly different anti-biofilm effect compared to the others. These results suggest that the extent of biofilm inhibition varied depending on the treatment applied, whether administered individually or in combination. 

**Table 4:** One-way ANOVA results for the OD630 values of the biofilm assay Note: "-" indicates the absence of corresponding data.

Source	df	Sum Sq	Mean Sq	F value	Pr(>F)
Condition	18	3.0563	0.1698	438.2151	<2 x 10^-16^
Residuals	38	0.0147	4.00 x 10^-4^	-	-

The significant differences observed among treatment groups may be attributed to variations in the presence and interaction of bioactive compounds derived from *T. catappa* and ciprofloxacin. Differences in OD₆₃₀ values reflect changes in bacterial adhesion, biofilm maturation, and extracellular polymeric substances (EPS) production, which are critical processes in biofilm development. 

Pairwise comparisons using Tukey's honest significant difference (HSD) post-hoc test for the biofilm assay are shown in Table 5. In Tukey's post-hoc analysis, p-values were adjusted due to multiple pairwise comparisons and to account for the possibility of committing a type I error (false positives), hence controlling the family-wise error rate at α = 0.05. The post-hoc test yielded 171 pairwise comparisons, of which only 24 had insignificant results. All extracts, as single agent or in combination, are significantly different from the negative and solvent, indicating that their inhibitory properties in this assay are significantly different from the baseline. The table displays only the non-significant pairs because these comparisons highlight treatment conditions that yield similar anti-biofilm effects despite differences in extract concentration or ciprofloxacin dosage. Identifying which combinations are statistically indistinguishable is equally important as identifying those that differ, as it reveals potential equivalent efficacy zones, plateau effects, or concentration ranges where increasing dosage does not yield additional inhibitory benefit.

**Table 5:** Tukey's honest significant difference (HSD) post-hoc rest for biofilm assays

Comparison	Diff	Lower Cl	Upper CI	Adjusted p-value
(Comb) Extract 16 + Cip 0.03125-(Comb) Extract 16 + Cip 0.015631	-0.0593	-0.1199	0.0013	0.0605
(Comb) Extract 16 + Cip 0.0625-(Comb) Extract 16 + Cip 0.03125	-0.0367	-0.0973	0.0239	0.7134
(Comb) Extract 32 + Cip 0.015625-(Comb) Extract 16 + Cip 0.0625	-0.0433	-0.1039	0.0173	0.4379
(Comb) Extract 32 + Cip 0.03125-(Comb) Extract 32 + Cip 0.015625	-0.051	-0.1116	0.0096	0.1913
(Comb) Extract 32 + Cip 0.0625-(Comb) Extract 32 + Cip 0.03125	-0.0283	-0.0889	0.0323	0.949
(Comb) Extract 64 + Cip 0.015625-(Comb) Extract 32 + Cip 0.03125	-0.0537	-0.1143	0.0069	0.1356
(Comb) Extract 64 + Cip 0.015625-(Comb) Extract 32 + Cip 0.0625	-0.0253	-0.0859	0.0353	0.9813
(Comb) Extract 64 + Cip 0.03125-(Comb) Extract 64 + Cip 0.015625	-0.0437	-0.1043	0.0169	0.4248
(Comb) Extract 64 + Cip 0.0625-(Comb) Extract 64 + Cip 0.03125	-0.0383	-0.0989	0.0223	0.6457
Cip 0.015625 ug/mL (1/8 MIC)-(Comb) Extract 32 + Cip 0.015625	-0.0503	-0.1109	0.0103	0.2076
Cip 0.015625 ug/mL (1/8 MIC)-(Comb) Extract 32 + Cip 0.03125	7.0 x 10^-4^	-0.0599	0.0613	1
Cip 0.015625 ug/mL (1/8 MIC)-(Comb) Extract 32 + Cip 0.0625	0.029	-0.0316	0.0896	0.9383
Cip 0.015625 ug/mL (1/8 MIC)-(Comb) Extract 64 + Cip 0.015625	0.0543	-0.0063	0.1149	0.124
Cip 0.03125 ug/mL (1/4 MIC)-(Comb) Extract 16 + Cip 0.015631	0.015	-0.0456	0.0756	1
Extract 32 ug/mL (1/4 MIC)-(Comb) Extract 16 + Cip 0.03125	-0.054	-0.1146	0.0066	0.1297
Extract 32 ug mL (1/4 MIC)-(Comb) Extract 16 + Cip 0.0625	-0.0173	-0.0779	0.0433	0.9998
Extract 32 ug/mL (1/4 MIC)-(Comb) Extract 32 + Cip 0.015625	0.026	-0.0346	0.0866	0.9761
Extract 64 ug/mL (1/2 MIC)-(Comb) Extract 32 + Cip 0.0625	-0.0507	-0.1113	0.0099	0.1993
Extract 64 ug/mL (1/2 MIC)-(Comb) Extract 64 + Cip 0.015625	-0.0253	-0.0859	0.0353	0.9813
Extract 64 ug/mL (1/2 MIC)-(Comb) Extract 64 + Cip 0.03125	0.0183	-0.0423	0.0789	0.9995
Extract 64 ug/mL (1/2 MIC)-(Comb) Extract 64 + Cip 0.0625	0.0567	-0.0039	0.1173	0.0895
Positive control (Ciprofloxacin 0.125 ug/mL + bacteria)-(Comb) Extract 16 + Cip 0.015631	0.015	-0.0456	0.0756	1
Positive control (Ciprofloxacin 0.125 ug/ml + bacteria) -Cip 0.03125 19/mL (1/4 MIC)	0	-0.0606	0.0606	1

Looking at the post-hoc pairs in the table, within each extract concentration, the different ciprofloxacin concentrations combined with the extract did not differ significantly from one another. As an example, the extract at 16 µg/mL combined with ciprofloxacin at 0.015625, 0.03125, and 0.0625 µg/mL showed no significant differences from each other, and the same pattern can be observed at higher concentrations of the extract. Similarly, the extract alone at a certain dose does not significantly differ from the same dosage of the extract combined with varying concentrations of ciprofloxacin. For example, the extract at 64 µg/mL is not significantly different from the same extract combined with varying concentrations of ciprofloxacin. These suggest that changing the ciprofloxacin concentration at a fixed dose of the extract does not vary the inhibitory capability of the combination that much. A significant difference was only observed when comparing different doses of the extract, which indicates a dose-dependent relationship. 

Notably, it can be seen that positive control did not significantly differ from low-dose concentrations (ciprofloxacin at 0.03125 µg/mL, extract 16 + ciprofloxacin 0.015631). The positive control is not superior to several combination treatments, showing that synergistic or additive effects may allow lower doses to match its inhibition. 

Quorum-sensing activity of *T. catappa* leaf extract

The swimming and swarming motility measurement and percentage reductions for each treatment condition are summarized in Table 6. To evaluate the quorum-sensing inhibitory property of *T. catappa* and its combination with ciprofloxacin, both swimming and swarming motility assays were conducted. A smaller motility zone compared to the control will be taken as an indication of quorum-sensing inhibition. The motility assays included a complete set of controls to ensure that any observed decrease in movement could be correctly attributed to quorum-sensing interference. The percentage reduction was calculated relative to the negative control.

**Table 6:** Quorum-sensing inhibitory activity of Terminalia catappa extract and ciprofloxacin (swimming vs. swarming)

Condition	Swimming		Swarming	
	Motility (mm)	% Reduction	Motility (mm)	% Reduction
Solvent control (1% DMSO)	28.67 ± 1.15	4.44 ± 3.85	53.67 ± 0.58	2.42 ± 1.05
Negative control (bacteria only)	30.00 ±1.00	0.00 ± 3.33	55.00 ± 1.00	0.00 ±1.82
Cip 0.015625 µg/mL (1/8 MIC)	26.67 ± 1.15	11.11 ± 3.85	52.00 ± 1.00	5.45 ± 1.82
Cip 0.03125 µg/mL (1/4 MIC)	23.00 ± 1.00	23.33 ± 3.33	46.67 ± 1.53	15.15 ± 2.78
Cip 0.0625 µg/mL (1/2 MIC)	19.33 ± 2.08	35.56 ± 6.94	39.67 ± 1.15	27.88 ± 2.10
Extract 16 µg/mL (1/8 MIC)	25.67 ± 1.53	14.44 ± 5.09	50.00 ± 1.00	9.09 ± 1.82
Extract 32 µg/mL (1/4 MIC)	21.67 ± 1.15	27.78 ± 3.85	44.67 ± 1.53	18.79 ± 2.78
Extract 64 µg/mL (1/2 MIC)	18.00 ± 1.00	40.00 ± 3.33	35.00 ± 1.00	36.36 ± 1.82
(Comb) Extract 16 + Cip 0.015631	25.67 ± 1.53	14.44 ± 5.09	48.33 ± 2.08	12.12 ± 3.78
(Comb) Extract 16 + Cip 0.03125	25.00 ± 1.00	16.67 ± 3.33	46.33 ± 0.58	15.76 ± 1.05
(Comb) Extract 16 + Cip 0.0625	23.67 ± 1.15	21.11 ± 3.85	44.33 ± 2.08	19.39 ± 3.78
(Comb) Extract 32 + Cip 0.015628	22.00 ± 1.00	26.67 ± 3.33	41.67 ± 0.58	24.24 ± 1.05
(Comb) Extract 32 + Cip 0.03125	20.00 ± 1.00	33.33 ± 3.33	39.67 ± 1.53	27.88 ± 2.78
(Comb) Extract 32 + Cip 0.0625	17.67 ± 1.53	41.11 ± 5.09	38.00 ± 1.00	30.91 ± 1.82
(Comb) Extract 64 + Cip 0.015625	19.00 ± 1.00	36.67 ± 3.33	35.67 ± 1.15	35.15 ± 2.10
(Comb) Extract 64 + Cip 0.03125	16.67 ± 1.15	44.44 ± 3.85	32.33 ± 2.08	41.21 ± 3.78
(Comb) Extract 64 + Cip 0.0625	15.00 ± 1.00	50.00 ± 3.33	30.00 ± 1.00	45.45 ± 1.82

Across all treatments, both swimming and swarming motility reduce as either ciprofloxacin or the extract concentration increases. The negative control exhibited the highest motility at 30% for swimming and 55% for swarming, which is expected since this is the baseline. Similarly, the solvent also had high motility, which established that the vehicle did not interfere with motility. 

 Both single agents (ciprofloxacin, *T. catappa*) and a combination of these exhibited dose-dependent reduction in motility for both swimming and swarming. That is, there is maximum reduction at the maximum dose tested for a specific condition (single and combination).

Overall, the motility data demonstrate that dose impacts the performance of both the crude *T. catappa* extract and ciprofloxacin in terms of quorum-sensing inhibitory activity.

**Figure 3:**
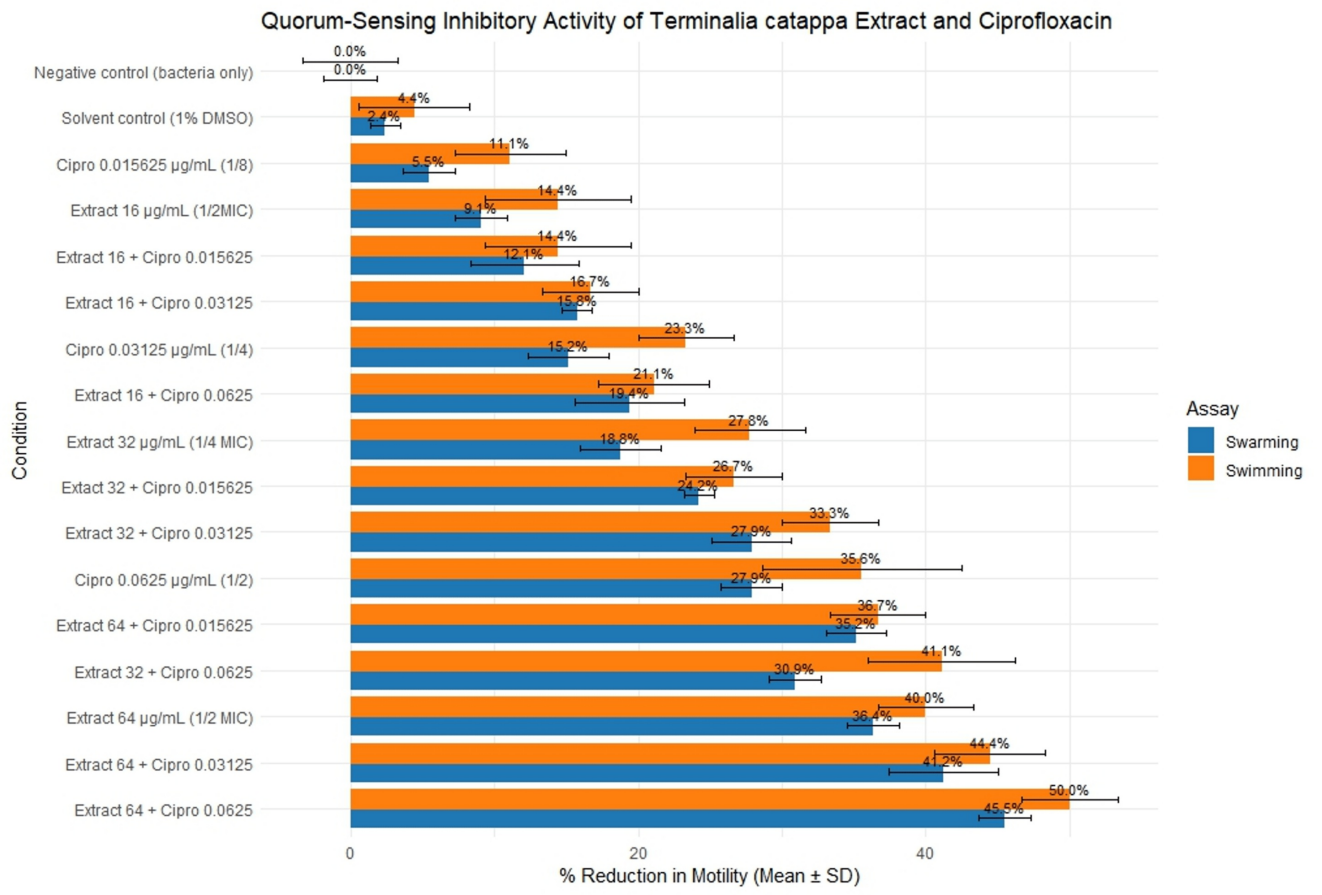
Effects of sub-MIC concentrations of Terminalia catappa crude leaf extract, ciprofloxacin, and their combinations on quorum sensing-regulated swarming and swimming motility of Pseudomonas aeruginosa BIOTECH 1335 Quorum sensing-regulated motility inhibition of *Pseudomonas aeruginosa* following treatment with sub-MIC concentrations of *Terminalia catappa* crude leaf extract, ciprofloxacin, and their combinations. Blue bars represent swarming motility, and orange bars represent swimming motility. Bars indicate mean percentage reduction in motility, and error bars denote standard deviation (SD). Negative control (bacteria only) and solvent control (1% DMSO) are included for comparison. Image created by the authors with Microsoft Excel (Microsoft Corp., USA)

The effects of sub-MIC concentrations of the extract, ciprofloxacin, and their combinations on quorum-sensing-regulated swimming and swarming motility are illustrated in Figure 3. The grouped bar plot demonstrates the percentage of reduction in motility on both swimming and swarming across the different conditions in the study, and is arranged accordingly as well. The plot shows a clear dose-dependent trend in quorum-sensing inhibition for both swimming and swarming. 

Expectedly, the least reduction, as mentioned earlier, was the negative and the solvent control, which posed negligible reductions in motility; baseline movement is not affected in the absence of any treatment. As ciprofloxacin and *T. catappa* ethanolic leaf extract were introduced, a modest reduction in motility was observed, and its effect strengthened as its concentration increased. The lowest reduction among the conditions is from the lowest dose of ciprofloxacin at 0.015625 µg/mL, and this is for both swimming and swarming. Following this is the smallest sub-MIC dose tested for the *T. catappa* extract at 16 µg/mL. 

A combination of both ciprofloxacin and *T. catappa* ethanolic leaf extract and increasing dose of each agent results in a higher reduction in motility, indicating that combining these agents results in a greater reduction in motility for both swimming and swarming. 

In addition, across all conditions, swimming motility showed greater reduction in motility, indicating that swimming behavior is more sensitive to quorum-sensing interference relative to these conditions. Overall, the visualization clearly demonstrates a dose-dependent trend and how motility in swimming is more impacted by the reduction of motility. 

The results of the one-way ANOVA for swimming and swarming motility assays are presented in Table 7. At the 0.05 level of significance, statistically significant differences were observed among the conditions for both swimming and swarming motility, indicating that at least one condition exhibited a significantly different effect on motility.

**Table 7:** One-way ANOVA results for the motility values of quorum sensing Note: "-" indicates the absence of corresponding data.

Assay	Source	df	Sum Sq	Mean Sq	F value	Pr(>F)
Swimming	Condition	16	9.05 x 10^2^	56.5	37.0	7.56 x 10^-17^
	Residuals	34	52.0	1.53	-	-
Swarming	Condition	16	2.68 x 10^3^	1.68 x 10^2^	96.1	1.49 x 10-^23^
	Residuals	34	59.3	17.5	-	-

The results of Tukey's HSD post-hoc analysis for swimming motility are shown in Table 8, while the results for swarming motility are shown in Table 9. Post-hoc analysis using Tukey’s HSD test was subsequently performed to determine which specific treatment conditions differed significantly from one another.

**Table 8:** Tukey's honest significant difference (HSD) post-hoc test for swimming

Comparison	Diff	Lower CI	Upper CI	Adjusted p-value
Extract 64 + Cipro 0.0625-Cipro 0.015625 ug/mL (1/8)	-11.70	-15.40	-7.90	3.30 x10^-11^
Extract 64 + Cipro 0.0625-Cipro 0.03125 ug/mL (1/4)	-8.00	-11.80	-4.23	3.87 x 10^-7^
Extract 64 + Cipro 0.0625-Cipro 0.0625 ug/mL (1/2)	-4.33	-8.10	-0.57	1.21 x 10^-2^
Extract 64 + Cipro 0.0625-Extract 16 ug/mL (1/2MIC)	-10.70	-14.40	-6.90	3.61 x 10^-10^
Extract 64 + Cipro 0.0625-Extract 32 ug/mL (1/4 MIC)	-6.67	-10.40	-2.90	1.68 x 10^-5^
Extract 64 ug/mL (1/2 MIC)-Extract 64 + Cipro 0.0625	3.00	-0.77	6.77	0.25
Extract 64 + Cipro 0.0625-Extract 16 + Cipro 0.015625	-10.70	-14.40	-6.90	3.61 x 10^-10^
Extract 64 + Cipro 0.0625-Extract 16 + Cipro 0.03125	-10.00	-13.80	-6.23	1.91 x 10^-9^
Extract 64 + Cipro 0.0625-Extract 16 + Cipro 0.0625	-8.67	-12.40	-4.90	6.26 x 10^-8^
Extract 64 + Cipro 0.0625-Extact 32 + Cipro 0.015625	-7.00	-10.80	-3.23	6.47 x 10^-6^
Extract 64 + Cipro 0.0625-Extract 32 + Cipro 0.03125	-5.00	-8.77	-1.23	1.97 x 10^-3^
Extract 64 + Cipro 0.0625-Extract 32 + Cipro 0.0625	-2.67	-6.43	1.10	0.43
Extract 64 + Cipro 0.0625-Extract 64 + Cipro 0.015625	-4.00	-7.77	0.23	2.83 x 10^-2^
Extract 64 + Cipro 0.0625-Extract 64 + Cipro 0.03125	-1.67	-5.43	2.10	0.96

Two sets of post hoc tests were done for both the swimming and swarming assays. There were a total of 136 pairwise comparisons resulting from Tukey’s HSD post-hoc test. Because the full post-hoc test generated 136 pairwise comparisons, only the contrasts involving the treatment with the largest motility reduction (extract 64 µg/mL + ciprofloxacin 0.0625 µg/mL) were interpreted. Statistical guidelines emphasize that when the number of contrasts is very large, interpretation may be restricted to theoretically meaningful comparisons to avoid Type I error inflation and to retain biological relevance (Maxwell & Delaney, 2017) [16].

The post-hoc analysis against the top treatment shows that this combination produced significantly greater reduction in swimming motility as compared to ciprofloxacin-only conditions and extract-only conditions across all tested concentration doses of these agents. This finding indicates that at this combination, it achieved a level of swimming motility reduction that neither of the agents produced independently across all tested sub-inhibitory concentrations. 

When compared with lower doses of extract-ciprofloxacin combination, the extract 64 µg/mL + ciprofloxacin 0.0625 µg/mL consistently showed significantly stronger reduction in swimming motility. Significant differences were observed relative to the combinations containing extract at 16 µg/mL and 32 µg/mL paired with ciprofloxacin across all sub-inhibitory concentrations of this agent. Conditions that achieved similar swimming reduction capability as the extract 64 µg/mL + ciprofloxacin 0.0625 µg/mL (p > 0.05) are the same extract combined with ciprofloxacin at 0.03125 µg/mL, extract 32 µg/mL + ciprofloxacin 0.0625 µg/mL, and extract 64 µg/mL alone. These non-significant contrasts indicate that at the highest extract concentration, further addition of ciprofloxacin may not yield a substantial incremental reduction in swimming motility.

**Table 9:** Tukey's honest significant difference (HSD) post-hoc test for swarming assay

Comparison	Diff	Lower CI	Upper CI	Adjusted p-value
Extract 64 + Cipro 0.0625-Cipro 0.015625 ug/mL (1/8)	-22.00	-26.00	-18.00	<0.001
Extract 64 + Cipro 0.0625-Cipro 0.03125 ug/mL (1/4)	-16.70	-20.70	-12.60	<0.001
Extract 64 + Cipro 0.0625-Cipro 0.0625 ug/mL (1/2)	-9.67	-13.70	-5.64	<0.001
Extract 64 + Cipro 0.0625-Extract 32 + Cipro 0.015625	-11.70	-15.70	-7.64	<0.001
Extract 64 + Cipro 0.0625-Extract 16 + Cipro 0.015625	-18.30	-22.40	-14.30	<0.001
Extract 64 + Cipro 0.0625-Extract 16 + Cipro 0.03125	-16.30	-20.40	-12.30	<0.001
Extract 64 + Cipro 0.0625-Extract 16 + Cipro 0.0625	-14.30	-18.40	-10.30	<0.001
Extract 64 + Cipro 0.0625-Extract 16 ug/mL (1/2MIC)	-20.00	-24.00	-16.00	<0.001
Extract 64 + Cipro 0.0625-Extract 32 + Cipro 0.03125	-9.67	-13.70	-5.64	<0.001
Extract 64 + Cipro 0.0625-Extract 32 + Cipro 0.0625	-8.00	-12.00	-3.98	<0.001
Extract 64 + Cipro 0.0625-Extract 32 ug/mL (1/4 MIC)	-14.70	-18.70	-10.6	<0.001
Extract 64 + Cipro 0.0625-Extract 64 + Cipro 0.015625	-5.67	-9.69	-1.64	0.001
Extract 64 + Cipro 0.0625-Extract 64 + Cipro 0.03125	-2.33	-6.36	1.69	0.740
Extract 64 ug/mL (1/2 MIC)-Extract 64 + Cipro 0.0625	5.00	0.98	9.02	0.005

Findings from the post hoc test for the swarming assay produced similar results as the swimming assay. The result showed that extract 64 µg/mL + ciprofloxacin 0.0625 µg/mL produced the strongest reduction in swarming motility among all treatments. This combination was significantly more effective than all ciprofloxacin-only conditions (p < 0.001) and the majority of extract-only or lower-concentration extract-ciprofloxacin combinations, with mean differences ranging from 22 to 8 units. Only the extract 64 µg/mL + ciprofloxacin 0.03125 µg/mL had a similar swarming reduction capability as the top treatment. Overall, the swarming motility results parallel the swimming assay, establishing that the top treatment is from the combination of the extract and ciprofloxacin at their highest dose tested.

Synergistic effect of *T. catappa *leaf extract and ciprofloxacin

The interaction between *T. catappa *leaf extract and ciprofloxacin was assessed using a checkerboard assay. The resulting ∑FIC values and corresponding interactions are summarized in Table 10.

**Table 10:** Fractional inhibitory concentration index (∑FIC) values of Terminalia catappa crude leaf extract and ciprofloxacin combinations against Pseudomonas aeruginosa BIOTECH 1335

Extract (µg/mL)	Ciprofloxacin (µg/mL)	OD630 (Mean ± SD)	ΣFIC	Interaction
16	0.015625	0.028 ± 0.003	0.250	Synergistic
16	0.031250	0.036 ± 0.002	0.375	Synergistic
16	0.062500	0.102 ± 0.008	0.625	Additive
32	0.015625	0.038 ± 0.003	0.375	Synergistic
32	0.031250	0.052 ± 0.003	0.500	Synergistic
32	0.062500	0.116 ± 0.005	0.750	Additive
64	0.015625	0.115 ± 0.005	0.625	Additive
64	0.031250	0.131 ± 0.004	0.750	Additive
64	0.062500	0.195 ± 0.005	1.000	Additive

The checkerboard assay revealed that the synergistic effect between the *T. catappa* ethanolic leaf extract and ciprofloxacin occurred only at lower extract concentrations, 16 and 32 µg/mL, combined with sub-inhibitory ciprofloxacin doses (0.015625 µg/mL, 0.03125 µg/mL). By contrast, combinations involving higher extract concentrations (64 µg/mL) or ciprofloxacin at the highest sub-inhibitory concentration (0.0625 µg/mL) combined across all tested concentrations shifted towards additive interaction, indicating possible diminishing of the synergistic effect at these doses. Overall, the data suggest that the most effective synergistic concentrations are achieved with low-to-moderate extract doses (16-32 µg/mL) paired with low ciprofloxacin amounts (0.015625-0.03125 µg/mL), highlighting the potential of these combinations to enhance antimicrobial efficacy against *P. aeruginosa* BIOTECH 1335 while minimizing antibiotic exposure.

The results of the two-way ANOVA assessing the effects of extract and ciprofloxacin concentrations on OD_630 _values are presented in Table 11.

**Table 11:** Two-way ANOVA of OD630 by extract and ciprofloxacin Note: "-" indicates the absence of corresponding data.

Source of variation	Sum of squares	df	Mean square	F-value	p-value
Extract concentration (µg/mL)	4.42 x 10^-2^	2	2.21x 10^-2^	1136.402	<0.001
Ciprofloxacin concentration (µg/mL)	3.09 x 10^-2^	2	1.54 x 10^-2^	793.888	<0.001
Interaction	6.48 x 10^-5^	4	1.62 x 10^-5^	0.833	0.522
Residuals	3.50 x 10^-4^	18	1.94 x 10^-5^	-	-

Table 11 shows the results of the two-way analysis of variance (ANOVA) conducted to assess whether the observed differences in microbial growth, as measured by OD₆₃₀ absorbance, were attributable to the individual main effects of crude extract concentration and ciprofloxacin concentration, as well as to their interaction. The analysis demonstrated that both extract concentration and ciprofloxacin exerted statistically significant main effects on microbial growth ( p < 0.001 for both factors). In contrast, the interaction between extract and ciprofloxacin concentrations was not statistically significant, indicating that the combined effects of the two agents were predominantly additive rather than multiplicative within the tested concentration range. 

Accordingly, although synergistic inhibition was observed at selected concentration pairs in the checkerboard assay, the non-significant interaction term in the two-way ANOVA reflects the absence of a consistent interaction effect across all tested combinations. This highlights the distinction between localized synergy detected by checkerboard analysis and overall interaction trends evaluated using two-way ANOVA.

Post-hoc Tukey's HSD analysis of the main effects of extract and ciprofloxacin concentrations is shown in Table 12.

**Table 12:** Post-hoc Tukey honest significant difference (HSD) for the main effects

Treatment	Comparison	Mean Difference	Lower CI	Upper CI	p-value
Extract	32-16	0.01322	7.917 x 10^-3^	1.853 x 10^-2^	1.554 x 10^-5^
	64-16	0.09167	8.636 x 10^-2^	9.697 x 10^-2^	2.531 x 10^-14^
	64-32	0.07844	7.314 x 10^-2^	8.375 x 10^-2^	2.531 x 10^-14^
Ciprofloxacin	0.03125-0.015625	1.267 x 10^-2^	7.361 x 10^-3^	1.797 x 10^-2^	2.659 x 10^-5^
	0.0625-0.015625	7.722 x 10^-2^	7.192 x 10^-2^	8.253 x 10^-2^	2.531 x 10^-14^
	0.0625-0.03125	6.456 x 10^-2^	5.925 x 10^-2^	6.986 x 10^-2^	2.531 x 10^-14^

The non-significant interaction validates the use of post-hoc comparison focused on the main effects instead of all possible pairwise comparisons containing interaction effects. The post-hoc comparison assumes that we hold each other's main effect constant to assess their significance. The result shows significant differences across all tested sub-inhibitory concentrations of both ciprofloxacin and the *T. catappa* ethanolic leaf extract. The direction dictates that OD630 of higher doses is significantly lower than the lower doses tested. The result dictates a clear dose-dependent relationship for both agents alone.

## Discussion

This study evaluated the synergistic anti-quorum-sensing-associated and anti-biofilm activities of *T. catappa* ethanolic leaf extract in combination with ciprofloxacin against *P. aeruginosa* BIOTECH 1335. The findings demonstrate that the crude extract contains biologically relevant phytochemical constituents capable of modulating bacterial behavior at sub-inhibitory concentrations and that its combination with ciprofloxacin enhances antibacterial efficacy within defined concentration ranges.

The ethanolic extraction of fresh *T. catappa* leaves yielded 2.46%, confirming the successful recovery of extractable phytoconstituents. Although lower than yields reported in studies utilizing dried plant material or alternative solvents, this variation is expected due to differences in moisture content, solvent polarity, and extraction conditions [17,18]. Despite the relatively modest yield, the extract demonstrated functional bioactivity, indicating that extraction efficiency does not necessarily correlate with biological effectiveness.

Qualitative phytochemical screening revealed the presence of flavonoids, tannins, cardiac glycosides, reducing sugars, and fixed oils and fats, while alkaloids and anthraquinone glycosides were absent. These findings are consistent with previous reports identifying phenolic and flavonoid compounds as predominant constituents of *T. catappa* leaves [1,19]. Flavonoids and tannins are widely recognized for their antimicrobial and antivirulence properties, including interference with quorum-sensing pathways and inhibition of biofilm development, supporting their relevance to the observed biological effects in this study.

FTIR-ATR analysis further substantiated the phytochemical screening results by identifying functional groups characteristic of polyphenolic compounds. The presence of broad O-H stretching bands, carbonyl (C=O) groups, aromatic C=C vibrations, and C-O stretching modes indicates a complex mixture of phenolic and glycosidic structures. These functional groups have been associated with antimicrobial, antioxidant, and antibiofilm activities in previous investigations of *T. catappa* extracts [20,21]. The reproducibility of the FTIR spectra across independent trials also confirms the chemical stability of the extract.

The minimum inhibitory concentration of the *T. catappa* crude extract against *P. aeruginosa *BIOTECH 1335 was determined to be 128 µg/mL, while ciprofloxacin exhibited an MIC of 0.125 µg/mL. These values are comparable to previously reported MIC ranges for plant extracts and fluoroquinolone antibiotics against *P. *species [22,13]. Importantly, all subsequent biofilm and quorum-sensing-associated assays were conducted at sub-MIC concentrations, ensuring that the observed effects were not attributable to bactericidal activity but rather to interference with bacterial regulatory processes.

The crude extract demonstrated inhibitory effects on biofilm formation at sub-MIC concentrations, both when used alone and in combination with ciprofloxacin. Interestingly, ciprofloxacin exhibited an inverse dose-response pattern in the biofilm assay, wherein lower sub-MIC concentrations produced greater inhibition than higher concentrations. This atypical observation suggests that biofilm-associated responses to antibiotics may not always follow classical linear concentration-dependent trends and may reflect complex adaptive or regulatory behaviors within biofilm communities. These findings indicate that biofilm inhibition may involve multifactorial responses beyond direct bactericidal activity. 

Given the critical role of biofilms in antibiotic tolerance and persistent infections caused by *P. aeruginosa*, these findings are particularly significant [4]. Biofilm inhibition at non-lethal concentrations suggests that the extract may interfere with early stages of biofilm development, such as bacterial adhesion, extracellular polymeric substance production, or surface colonization. Similar antibiofilm effects have been reported for *T. catappa* extracts and related formulations in previous studies, although efficacy has been shown to vary depending on bacterial species and extract preparation [23,24].

Quorum-sensing-regulated swarming and swimming motility assays further demonstrated reduced motility in treated groups relative to controls, indicating disruption of quorum-sensing-associated phenotypes. In *P. aeruginosa*, motility behaviors are tightly regulated by quorum-sensing systems that also control virulence and biofilm formation [5]. The observed reduction in motility at sub-MIC concentrations supports the interpretation that the *T. catappa* extract may interfere with quorum-sensing-regulated behaviors rather than acting solely as a direct growth suppressant. This finding aligns with previous reports highlighting the potential of plant-derived polyphenols to attenuate bacterial communication and virulence without exerting strong selective pressure for resistance [25].

The checkerboard assay revealed synergistic and additive interactions between the *T. catappa* crude extract and ciprofloxacin, as indicated by fractional inhibitory concentration index values. Notably, synergistic interactions were primarily observed at lower extract (16-32 µg/mL) and sub-inhibitory ciprofloxacin concentrations, whereas higher-dose combinations demonstrated predominantly additive effects. Furthermore, several combination treatments involving higher extract concentrations were statistically comparable to the extract alone at the same concentration, suggesting a potential plateau effect. This indicates that maximal biofilm suppression may have already been achieved by the extract at higher doses, thereby limiting additional enhancement by ciprofloxacin under those conditions. These findings emphasize that the observed synergistic interaction was concentration-dependent rather than generalized across all tested combinations. Consistent with this observation, the non-significant interaction in the two-way ANOVA indicates that a uniform synergistic effect was not observed across all concentration levels, further supporting that the interaction between the extract and ciprofloxacin was dependent on specific dose combinations rather than broadly consistent across the experimental matrix.

Synergistic interactions between plant extracts and antibiotics have been widely documented and are often attributed to complementary mechanisms such as increased membrane permeability, inhibition of efflux pumps, disruption of quorum-sensing-regulated pathways, and weakening of biofilm architecture [6,26]. The present findings support this concept and demonstrate that *T. catappa* extract can function as an effective antibiotic adjuvant within defined concentration ranges.

It should be noted that phytochemical screening in this study was qualitative, and no minimum bactericidal concentration (MBC) was determined. Therefore, the bactericidal versus bacteriostatic nature of the extract and combination treatment could not be conclusively established. Additionally, quorum-sensing modulation was inferred from phenotypic assays rather than direct molecular measurements. The inverse dose-response pattern observed for ciprofloxacin in the biofilm assay was not mechanistically investigated in this study and therefore remains to be elucidated. Furthermore, the study was conducted using a single reference strain of *P. aeruginosa, *and validation across multiple clinical isolates would strengthen the broader applicability of these findings.

Collectively, the results of this study indicate that *T. catappa* ethanolic leaf extract possesses anti-biofilm and anti-quorum-sensing-associated modulatory properties and can enhance the activity of ciprofloxacin against *P. aeruginosa *under specific concentration conditions. By targeting bacterial communication and biofilm formation rather than relying solely on bactericidal effects, this combination strategy offers a promising adjunct approach for managing biofilm-associated and antibiotic-resistant infections. While the findings are limited to in vitro conditions, they provide a strong foundation for further mechanistic studies, toxicity evaluation, and potential translational research.

## Conclusions

Based on the findings, *T. catappa* ethanolic crude leaf extract contains important phytochemical constituents such as flavonoids and tannins that contribute to its antibacterial and anti-biofilm potential. The extract demonstrated dose-dependent anti-biofilm and quorum-sensing-associated modulatory effects against *P. aeruginosa *BIOTECH 1335 at sub-inhibitory concentrations.

When combined with ciprofloxacin, synergistic interactions were observed at specific concentration ranges, whereas higher-dose combinations exhibited predominantly additive or plateau effects. These results indicate that the interaction between the extract and ciprofloxacin is concentration-dependent rather than uniformly synergistic.

Although the in vitro findings support the potential of *T. catappa *extract as an antibiotic adjuvant under defined conditions, certain limitations should be considered, including the lack of chemical standardization, the absence of molecular validation of quorum-sensing inhibition, and evaluation using a single bacterial strain. Further studies addressing these aspects are warranted.

Overall, the results suggest that *T. catappa *extract may serve as a potential antibiotic adjuvant under defined concentration conditions, offering a complementary strategy for managing biofilm-associated infections.

## References

[1] Mwangi WC, Waudo W, Shigwenya ME, Gichuki J (2024). Phytochemical characterization, antimicrobial and antioxidant activities of Terminalia catappa methanol and aqueous extracts. BMC Complement Med Ther.

[2] Sowmya Sowmya, Raveesha K (2021). Antibacterial activity and time-kill assay of Terminalia catappa l. and Nigella sativa l. against selected human pathogenic bacteria. J Pure Appl Microbiol.

[3] Munira M, Miko A, Nasir M (2022). The Mixing of Ethanol Extract of Terminalia catappa L in Transparent Soap Base to maintaining it’s the Organoleptic properties, Solid quality and Inhibition of Staphylococcus aureus. Res J Pharm Technol.

[4] Haidar A, Muazzam A, Nadeem A (2024). Biofilm formation and antibiotic resistance in Pseudomonas aeruginosa. Microbe.

[5] Vashistha A, Sharma N, Nanaji Y, Kumar D, Singh G, Barnwal RP, Yadav AK (2023). Quorum sensing inhibitors as therapeutics: bacterial biofilm inhibition. Bioorg Chem.

[6] Atta S, Waseem D, Fatima H, Naz I, Rasheed F, Kanwal N (2023). Antibacterial potential and synergistic interaction between natural polyphenolic extracts and synthetic antibiotic on clinical isolates. Saudi J Biol Sci.

[7] Ghanad A (2023). An overview of quantitative research methods. Int J Multidiscipl Res Anal.

[8] Zubair A (2023). Experimental research esign-types & process. https://www.researchgate.net/publication/367044021_Experimental_Research_Design-types_process.

[9] Madhavan K, Rukayadi Y, Mutalib NaM (2023). Phytochemical constituents and toxicity analysis of ethanolic ketapang (Terminalia catappa l.) leaf extract. Malays Appl Biol.

[10] Shaikh JR, Patil M (2020). Qualitative tests for preliminary phytochemical screening: an overview. Int J Chem Stud.

[11] Vingadassalon A, Pejcz E, Wojciechowicz-Budzisz A (2024). Terminalia catappa Kernel Flour Characterization as a Functional and Bioactive Ingredient for Cookies Formulation. App Sci.

[12] Abdullah MI, Yahya MF, Bakar LM (2024). Efficacy of Terminalia catappa leaves extract as an antimicrobial agent against pathogenic bacteria. Malays Appl Biol.

[13] Liu Y, Moore JH, Kolling GL, McGrath JS, Papin JA, Swami NS (2020). Minimum bactericidal concentration of ciprofloxacin to Pseudomonas aeruginosa determined rapidly based on pyocyanin secretion. Sens Actuators B Chem.

[14] Dey P, De R, Parai D (2024). Enhanced antimicrobial activity of naringin-ciprofloxacin combination against Pseudomonas aeruginosa PAO1: unveiling quorum-sensing mediated molecular mechanisms in biofilm formation and virulence. Microbe.

[15] Nikolic I, Vukovic D, Gavric D (2022). An optimized checkerboard method for phage-antibiotic synergy detection. Viruses.

[16] Maxwell SE, Delaney HD, Kelley K (2017). Designing experiments and analyzing data: a model comparison perspective. https://www.taylorfrancis.com/books/mono/10.4324/9781315642956/designing-experiments-analyzing-data-scott-maxwell-harold-delaney-ken-kelley.

[17] Mantaring SD, Santos JR, Estrella R (2023). Terminalia catappa L. leaf extract interferes with biofilm formation of Vibrio parahaemolyticus and enhances immune response of Penaeus vannamei against acutehepatopancreatic necrosis disease. Aquaculture.

[18] Barde A, Oloyede R, Haruna A (2025). Phytochemical analysis and antibacterial activity of acetone extract of Terminalia catappa Linn. leaves. Trop J Drug Res.

[19] Orillaneda KN, Caracal E, Del Campo ID (2022). Phytochemical screening, antioxidant, and antibacterial property of talisay (Terminalia catappa) leaves ethanolic extract against Staphylococcus aureus used as formulated ointment. Int J Pharm Med Biol Sci.

[20] Kumar VD (2021). Phytochemical profiles, in vitro antioxidant, anti inflammatory and antibacterial activities of aqueous extract of Terminalia catappa L. leaves. J Pharm Sci Res.

[21] Bouagnon JJ, Konan Y, Sinan KI (2024). In vitro research to evaluate the antioxidant effects, inhibiting enzymes, methicillin-resistant Staphylococcus aureus strains of Terminalia catappa extracts. Sci Afr.

[22] Allyn OQ, Kusumawati E, Nugroho RA (2018). Antimicrobial activity of Terminalia catappa brown leaf extracts against Staphylococcus aureus ATCC 25923 and Pseudomonas aeruginosa ATCC 27853. F1000Res.

[23] Ramsundar K, Jain RK, Pitchaipillai SG (2023). Anti-quorum sensing of Terminalia catappa and Murraya koenigii against Streptococcus mutans. Cureus.

[24] Ansari MA, Kalam A, Al-Sehemi AG (2021). Counteraction of biofilm formation and antimicrobial potential of Terminalia catappa functionalized silver nanoparticles against Candida albicans and multidrug-resistant gram-negative and gram-positive bacteria. Antibiotics (Basel).

[25] Sionov RV, Steinberg D (2022). Targeting the holy triangle of quorum sensing, biofilm formation, and antibiotic resistance in pathogenic bacteria. Microorganisms.

[26] Sharif SA, Ismaeil AS, Ahmad AA (2020). Synergistic effect of different plant extracts and antibiotics on some pathogenic bacteria. Sci J Univ Zakho.

